# A Practical Guide to the Therapy of Narcolepsy and Hypersomnia Syndromes

**DOI:** 10.1007/s13311-012-0150-9

**Published:** 2012-10-11

**Authors:** Emmanuel J. M. Mignot

**Affiliations:** Stanford Center for Sleep Sciences and Medicine, Stanford University Medical School, Palo Alto, CA 94304 USA

**Keywords:** Narcolepsy, Hypocretin, Orexin, Sodium oxybate, Modafinil, Venlafaxine

## Abstract

Narcolepsy and other syndromes associated with excessive daytime sleepiness can be challenging to treat. New classifications now distinguish narcolepsy/hypocretin deficiency (also called type 1 narcolepsy), a lifelong disorder with well-established diagnostic procedures and etiology, from other syndromes with hypersomnolence of unknown causes. Klein-Levin Syndrome, a periodic hypersomnia associated with cognitive and behavioral abnormalities, is also considered a separate entity with separate therapeutic protocols. Non hypocretin-related hypersomnia syndromes are diagnoses of exclusion. These diagnoses are only made after eliminating sleep deprivation, sleep apnea, disturbed nocturnal sleep, and psychiatric comorbidities as the primary cause of daytime sleepiness. The treatment of narcolepsy/hypocretin deficiency is well-codified, and involves pharmacotherapies using sodium oxybate, stimulants, and/or antidepressants, plus behavioral modifications. These therapies are almost always needed, and the risk-to-benefit ratio is clear, notably in children. Detailed knowledge of the pharmacological profile of each compound is needed to optimize use. Treatment for other syndromes with hypersomnolence is more challenging and less codified. Preferably, therapy should be conservative (such as modafinil, atomoxetine, behavioral modifications), but it may have to be more aggressive (high-dose stimulants, sodium oxybate, etc.) on a case-by-case, empirical trial basis. As cause and evolution are unknown in these conditions, it is important to challenge diagnosis and therapy over time, keeping in mind the possibility of tolerance and the development of stimulant addiction. Kleine-Levin Syndrome is usually best left untreated, although lithium can be considered in severe cases with frequent episodes. Guidelines are provided based on the literature and personal experience of the author.

**Electronic supplementary material:**

The online version of this article (doi:10.1007/s13311-012-0150-9) contains supplementary material, which is available to authorized users.

## Introduction

According to the *Diagnostic and Statistical Manual of Mental Disorders, 5th edition* (DSM-5) currently being finalized, syndromes with primary hypersomnolence can be practically divided into 3 groups: 1) narcolepsy caused by hypocretin (orexin) deficiency, a disorder associated with Human Leukocyte Antigen (HLA) marker DQB1*06:02 and believed to be autoimmune (almost all cases with cataplexy), 2) Kleine-Levin Syndrome (KLS), and 3) syndromes with hypersomnolence unexplained by hypocretin abnormalities (generally without cataplexy) [1]. This last group is the most challenging and the most frequent diagnosis.

A similar revised *International Sleep Disorder Classification, 3rd edition* (ICSD3) is also being finalized and will use a similar framework to that of the DSM-5 with minor differences. In the ICSD3, narcolepsy caused by hypocretin deficiency is called “type 1 narcolepsy” while other hypersomnias (not likely due to hypocretin abnormalities) will stay subdivided into “type 2 narcolepsy” in the presence of a positive Multiple Sleep Latency Test (MSLT) with multiple Sleep Onset REM periods (SOREMPs) vs idiopathic hypersomnia otherwise (for more detail see American Psychiatric Association [1]).

In this publication, we will use the DSM-5 classification as a guideline because there is no evidence that the pathophysiology or therapeutic response is substantially different for hypersomnia with or without SOREMPs on the MSLT. First we will review the most commonly used medications across all conditions. Treatment specificities in children and for each of the three conditions will be discussed last.

## Amphetamines

**Fig. 1:**
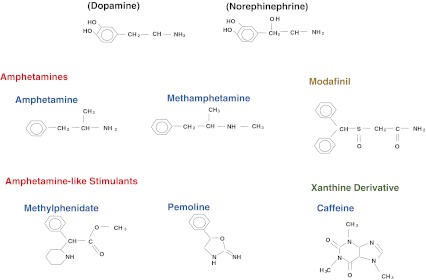
Chemical structures of amphetamine-like stimulants, modafinil, and caffeine (a xanthine derivative), as compared to dopamine and norepinephrine

Amphetamines are simple derivatives of catecholamines (dopamine, norepinephrine, epinephrine) that are made more lipophilic so that they enter the central nervous system easily (Fig. 1). These are very old chemical entities, first made available in 1935 [2]. These compounds mimic many of the catecholaminergic actions in the brain, primarily substituting for monoamines in presynaptic synapses and producing monoaminergic release [3] (Table 1).

**Table 1:** Commonly Used Available Narcolepsy Treatments and Their Pharmacological Properties

Compound	Pharmacological properties
Stimulants	
Amphetamine	Increases monoamine release (DA > NE> > 5-HT). Primary effects due to reverse efflux of DA through the DA transporter (DAT). Inhibition of monoamine storage through the vesicular monoamine transporter (VMAT) and other effects occur at higher doses. The D-isomer is more specific for DA transmission and is a better stimulant compound. Some effects on cataplexy (especially for the L-isomer), secondary to adrenergic effects, occur at higher doses. Available as racemic mixture or as pure D-isomer; various time-release formulations. Addiction potential is high for immediate-release formulation. Increased blood pressure and possible cardiac complications with high doses
Methamphetamine	Profile similar to amphetamine, but more lipophilic with increased central penetration, thanks to the addition of a methyl group. Now only available as immediate-release formulation. High addiction potential
Methylphenidate	Blocks monoamine (DA > NE> > 5-HT) uptake. No effect on reverse efflux or on VMAT. Short half-life. Available as racemic mixture or as pure D-isomer and in various time-release formulations. Addiction potential notable for immediate-release formulation
Selegiline	Also called L-desprenyl. monoamine oxidase B inhibitor with *in vivo* conversion into L-amphetamine and L-metamphetamine
Modafinil*	Fewer peripheral side effects. Low potency compound. Mode of action debated, but probably involves relatively selective DA reuptake inhibition. Available as a racemic mixture. Little, if any, addictive potential, but less efficacious than amphetamine or methylphenidate. The R-isomer has a longer half-life, and a similar profile to the racemic mixture and dosing differs (twice as strong)
Anti-cataplectic compounds	
Protriptyline	Tricyclic antidepressant. Monomoaminergic uptake blocker (NE > 5-HT > DA). Anticholinergic effects; all antidepressants have immediate effects on cataplexy, but abrupt cessation of treatment can induce very severe rebound in cataplexy
Clomipramine	Tricyclic antidepressant. Monoaminergic uptake blocker (5-HT > NE> > DA). Anticholinergic effects. Desmethyl-clomipramine (NE> > 5-HT > DA) is an active metabolite. No specificity *in vivo*
Venlafaxine*	Specific serotonin and adrenergic reuptake blocker (5-HT ≥ NE); very effective but some nausea and gastric upset. May have less sexual side effects than other antidepressants. Slightly stimulant, short half-life, extended-release formulation preferred
Duloxetine*	Similar to venlafaxine, but more potent and longer half-life. Rare hepatotoxicity
Atomoxetine*	Specific adrenergic reuptake blocker (NE) normally indicated for attention deficit hyperactivity. Slightly stimulant, short half-life and reduces appetite
Fluoxetine	Specific serotonin uptake blocker (5-HT> > NE = DA). Active metabolite norfluoxetine has more adrenergic effects. High therapeutic doses are often needed
Other	
Sodium oxybate*	May act via GABA-B or specific GHB receptors. Reduces DA release. Need at minimum bi-nightly dosing with immediate effects on disturbed nocturnal sleep; therapeutic effects on cataplexy and daytime sleepiness can be delayed weeks to months. Nausea, weight loss, and psychiatric complications are possible side effects. As for any sedative, use with caution in the presence of hypoventilation or sleep apnea

5-HT = serotonin; DA = dopamine; MAO = monoamine oxidase; NE = norepinephrine; GABA-B = Gamma-aminobutyric acid receptor type B; GHB=Gamma-hydroxybutyric acid

*Compounds that could be considered as first intention treatments in narcolepsy/cataplexy, considering their benefit/side effect profiles when compared to other medications

Amphetamine derivatives not only affect adrenergic and dopaminergic synapses [4], but also to a lesser extent serotoninergic synapses; specificity for each monoamine varies if further modifications are made (3,4-methylenedioxy-*N*-methylamphetamine [MDMA], a drug of abuse also known as Ecstasy, for example, has more of a serotonin effect).

As a rule, amphetamine isomers of the D-type are more active than isomers of the L-type, and have more effects on dopaminergic synapses than on other monoaminergic synapses [5]. L and D isomers of any derivatives may also have different pharmacokinetic properties. The addition of another methyl group to amphetamine creates methamphetamine, further increasing brain penetration and potency (Fig. 1). Effective doses are listed in Table 2, keeping in mind that there is great variation in effectiveness across formulations, and that it is unusual and probably not as safe to have to go beyond 60 mg/day.

**Table 2:** Therapeutic Protocols for Commonly Prescribed Narcolepsy Treatments

Compound	
Stimulants	
Modafinil	As a racemic mixture available as 100 and 200 mg, administered once or twice a day (morning and noon), with a maximum of 400 mg/day as typical. Also available as R-modafinil (50, 150, and 250 mg), typically approximately twice more potent than racemic modafinil per mg, once steady state concentration is achieved. Headache is a common side effect but is usually avoidable by increasing the dose slowly. Possible allergic side effects to monitor, notably in children when it has not been Food and Drug Administration approved
Methylphenidate	More effective and potent than modafinil and low cost. Can substitute for modafinil when using long-acting formulations of the racemic mixture of any single isomer typically 20 to 40 mg/day. Various preparations and formulations can have substantially different interindividual effects. As base preparation, immediate release (5–10 mg) can be helpful, either to alleviate sleep drunkenness in hypersomnia, to bridge gaps in alertness during the daytime (postprandial dose), or to use when necessary in case of emergency (e.g. need to drive to the hospital)
Amphetamine	Use similar to methylphenidate, preferably use long-lasting formulations. Can be more effective than methylphenidate in some patients. Monitoring of pulse and blood pressure needed
Anti-cataplectic compounds	
Clomipramine	Low cost and extemely effective. Is immediately effective on cataplexy as all antidepressants. Can be very effective at doses as low as 25 mg/day in the morning or evening, but more typically 75 mg/day is needed, with a maximum of 150 mg/day. Side effects include dry mouth, sweating, constipation, blurred vision, and orthostatic hypotension. Notably contraindicated in subjects with cardiovascular conduction abnormalities. Tricyclic antidepressants must not be used in open-angle glaucoma.
Venlafaxine	Effective dose usually 37.5 to 150 mg/day (maximum, 300 mg/day). Slightly stimulant, short half-life, extended-release formulation preferred. Possible psychiatric withdrawal side effects with abrupt cessation (such as sudden electric shock-like feelings) and rebound cataplexy always present, unless also treated by other anti-cataplectic agents (such as sodium oxybate as a cover). Daily, scrupulous compliance needed. Gastrointestinal upset is a main reason for discontinuation. Transient constipation. Low available dosage useful for children
Duloxetine	Similar to venlafaxine, but more potent and longer half-life. Effective doses 20 to 40 mg (maximum, 60 mg/day). Rare hepatotoxicity, so avoid if alcohol abuse
Atomoxetine	Slightly stimulant and also anti-cataplectic, so it can be used for the treatment of either cataplexy or sleepiness. Short half-life, thus generally needs two daily administrations. Effective doses 10 to 60 mg (maximum, 80 mg/day). Reduces appetite. Urinary retention is a possible side effect. Monitoring of pulse and blood pressure needed. Low available dosage useful for children
Fluoxetine	Because of its very long half-life, it allows more stable therapy. High therapeutic doses are often needed for cataplexy. Can also be useful if concomitant anxiety disorder
Active on all narcolepsy symptoms	
Sodium oxybate	Administration at night, effective in consolidating sleep in patients with disturbed sleep due to insomnia, excessive activity during rapid eye movement sleep, hypnagogic hallucinations and sleep paralysis. Effect on cataplexy and daytime sleepiness is evident soon after treatment begins, but builds further along several weeks
	In adults or adolescents, we advise starting with 4.5 g/night divided into two doses (bed time and middle of the night). In prepubertal children, typically 3 g/night is then increased to 6 to 9 g/night in most cases (4.5–6 g most often in prepubertal children). Time of administration may be switched to 1 to 2 h after bedtime for the first dose. Total dose may be also be distributed into three unequal doses, titrated to the needs of each patient, with the goal of ensuring best consolidated sleep with age appropriate amounts, also reducing dose-dependent side effects (enuresis, occasionally parasomnia)
	Most common side effects are nausea and loss of appetite, usually beneficial, but occasionally leading to reduced weight that becomes problematic. Psychiatric side effects possible, notably in patients with an anxious premorbid personality; specific serotonin uptake blocker can occasionally be added to mitigate these

Amphetamines have complex pharmacological effects that are dose dependent and not fully characterized [6]. Amphetamines substitute for monoamines at the level of the vesicular monoamine transporter (VMAT), disrupting granular storage of monoamine through a reserpine-like effect and increasing cytosolic dopamine [7]. Next, they substitute for monoamines at the level of monoaminergic transporters (notably the dopamine reuptake site [DAT]), and not only inhibit reuptake, but also enter the presynaptic synapse while creating a paradoxal reverse efflux of monoamines through the same transporter [3, 6, 7]. Finally, amphetamines can also inhibit the monoamine oxidase (MAO) enzymes at very high doses. These complex actions are not without consequences, as high doses of amphetamines are cytotoxic for monoaminergic neurons in animals [6, 7], an effect mostly documented with MDMA and serotoninergic synapses, but also present with regular amphetamines and dopaminergic transmission [7, 8]. The stepwise recruitment of additional effects on monoamine transmission likely explains the high efficacy of these compounds. Amphetamines are exceptionally wake-promoting, and at high doses also reduce cataplexy in narcoleptic patients [9], an effect best explained by its action on adrenergic and serotoninergic synapses.

Amphetamines are water-soluble (thus risk of diversion to intravenous administration) and very potent wake-promoting compounds. The pharmacokinetics for each isomer is different, with a primarily urinary elimination that is favored at high urinary pH, decreasing half-life. As for other stimulants (i.e., methylphenidate and modafinil), the formulation and type of salt used in each preparation is almost as important as the milligram dosage and isomer composition. Notably, due to its use in attention deficit hyperactivity disorder (ADHD), a countless number of slow or extended-release formulations are available [10, 11], increasing duration of action from a few hours (3-6 for D-amphetamine) to 12 hours or more. Slow-release formulations also slow down absorption, reducing peak concentration after administration, a property that reduces the “rush” or “quick” effects of nonextended-release formulation. The rapid rise in concentration, rush effects, and rapid on–off changes in dopamine released are likely important contributors to the addictive potential of amphetamines [12]. Amphetamines can be addictive, leading patients to feel unreasonably anxious or unwilling to stop, also seeking the drug at ever-increasing doses.

Numerous studies have shown that increased dopamine release is the main property explaining wake-promotion [3, 13], although norepinephrine effects also contribute. Side effects of amphetamines include peripheral release of norepinephrine, resulting in cardiac stimulation and vasoconstriction. Increased heart rate and blood pressure, palpitations, and sweating are common [14]. Increased anxiety can occur in predisposed patients. Mood may be temporarily enhanced, but the effect is generally not sustainable alone in patients with depression. At a high dose, an amphetamine may precipitate psychosis [15], although most typically delusions are of the persecution subtype and are reversible with cessation of the medication. As with all psychotropic medications, however, more severe and irreversible psychiatric complications can emerge.

Co-administration of an amphetamine with MAO inhibitors is contraindicated, as it can potentialize its effects, notably on blood pressure. Many MAO inhibitors are irreversible “suicide substrates” at MAO A and B enzymes that have an extremely long duration of action (weeks), and have minimal effects on wakefulness, making their use difficult to control. However, it is notable to remark that Selegiline (also called L-Desprenyl), a competitive MAO-B inhibitor that has been shown to be effective in narcolepsy, is primarily a metabolic precursor of amphetamine and exerts most of its therapeutic effects through amphetamine metabolism [16].

## Methylphenidate

Methylphenidate shares many of the actions of amphetamines, although it does not fully substitute for catecholamines at the level of the dopamine transporter. The compound became popular for use in narcolepsy in the 1950s [17]. It is primarily a DAT reuptake inhibitor, but it has also been shown to increase dopamine release [18, 19]. As an amphetamine, methylphenidate is also not fully specific for dopaminergic transmission, also affecting more mildly other monoamines. Furthermore, D-isomers have different pharmacokinetics and are more specific for dopaminergic synapses than the L-isomer. D-isomers are also more potent [20].

Methylphenidate has a short half-life and is water-soluble [21]. It is readily absorbed and enters the brain effectively, also producing rapidly increasing concentration in the brain and a sensation of rush. As for amphetamine, methylphenidate is addictive and can lead to drug-seeking behaviors and tolerance [18, 22]. Onset of effects after oral administration is 20 minutes and duration of action is only approximately three hours.

The addictive potential of methylphenidate is highly dependent on formulation, with extended-release formulations having less potential for addiction. Because it is soluble, prescribed methylphenidate can be reformulated differently and has a street value for stimulant abusers. The large number of formulations available for methylphenidate (D- (dextro), L- (levo), both, and various release formulations) as a treatment for ADHD has also increased choice for hypersomnia therapies [10, 11].

Methylphenidate has a similar side effect profile to amphetamines (e.g. cardiovascular effects, psychosis) [14], although as it is primarily a DAT inhibitor, it has not been shown to be as cytotoxic in animal studies. As for amphetamines, methylphenidate suppresses primarily non-REM sleep, promoting wakefulness, but also REM sleep. It is mostly effective on wakefulness and has minimal effects on cataplexy and other narcolepsy symptoms. Effective doses are listed in Table 1, keeping in mind that as for the amphetamines, there is great variation in effectiveness across preparations, and it is unusual to have to go beyond 60 mg/day, preferably using long-acting formulations.

## Modafinil

Modafinil was developed and first used for the treatment of narcolepsy in France in the 1980s. It has been studied in double-blind, placebo-controlled fashion in a large number of narcoleptic patients [23, 24] and is approved by the Food and Drug Administration (FDA) for narcolepsy, shift work disorder [25], and residual sleepiness in treated sleep apnea [26]. It was recognized as an active metabolite of adrafinil, a compound developed as a cognitive enhancer for the elderly. The compound is highly insoluble in water and has a low potency, meaning that active doses are in the range of hundreds of milligrams *vs* tens of milligrams for doses of amphetamine or methylphenidate that are needed to produce similar wake-promoting effects (equipotency). As a chemical entity, it is poorly and slowly absorbed due to the insolubility in water. Formulation is key, and when formulated as Provigil (racemic mixture of D- and L-modafinil), it is compounded into small beads that increase absorption and consistency of effects.

Because it was rapidly shown that modafinil is pharmacologically distinct from amphetamine (but less so from methylphenidate) in some animal tests, and that abuse potential for the compound was low, the mode of action of modafinil was touted as totally different from other stimulants, and did not involve dopamine [27, 28]. Indeed, after more than 20 years of experience with the compound, abuse with dependence remains rare, although there is no doubt that the compound is misused to increase performance rather than to treat disease in some cases [28]. In the late 1980s, studies at Stanford showed that the compound had very similar properties to very selective DAT reuptake inhibitors in a canine model of narcolepsy [29]. In particular, as for other DAT compounds, it was shown to reduce wakefulness without affecting REM sleep or reducing cataplexy, a symptom that in canine narcolepsy is mostly sensitive to adrenergic reuptake inhibition [3, 29].

This led us to test whether modafinil also binds the DAT transporter *in vitro* at concentrations consistent with its potent wake-promoting effects, something we demonstrated in 1994 [30, 31]. The debate on whether or not modafinil effects were due to Dopamine reuptake inhibition continued for many years, with most other scientists and the drug companies involved being unconvinced by this hypothesis. Of note, selective and high-affinity DAT inhibitors are not available for human use, as animal studies generally indicate high risk of abuse, and cocaine being a relatively specific DAT reuptake inhibitor. In 2001, together with Jonathan Wisor and Dale Edgar, we found that the wake-promoting effects of modafinil were completely abolished in DAT knockout mice [13], as are those of amphetamine-like stimulants and DAT reuptake inhibitors. In spite of these findings, most of those in the scientific community remained unconvinced. It was only when Nora Wolkov, in a series of experiments, found that modafinil administered at wake-promoting doses can displace DAT positron emission tomographic ligands *in vivo*, that the tide finally turned, with most investigators now agreeing with a primary dopaminergic mediation of modafinil wake-promoting effects [32, 33].

The fact that modafinil increases wakefulness through dopamine reuptake inhibition does not reduce its therapeutic value as a unique compound in the treatment of excessive daytime sleepiness. Indeed, positron emission tomography studies previously mentioned are increasingly showing that DAT occupation alone may not be sufficient for addiction [12]. Most notably, the speed at which DAT inhibitors enter the brain, leading to high brain peak concentrations with subsequent rapid decreases, may be most important, explaining why mode of administration is critical for abuse potential, with intravenous and smoking being the worst, followed by snorting. Oral administration of stimulants, especially as slow-release formulations, has lower abuse potential, even for amphetamine and methylphenidate. One likely hypothesis for the low risk of abuse with modafinil may thus be its very poor solubility and low potency, making it impossible to reformulate for adequate, high-speed brain delivery. As for any pharmaceutical entity, it is also possible that modafinil does not bind the DAT transporter in the same way as other DAT inhibitors [34], or that other unknown effects are involved, keeping with the pharmaceutical adage that any drug has effects that are known, and many that are unknown.

Modafinil is available as a racemic mixture, or as the R-isomer only. As L-modafinil has a lower affinity for the dopamine transporter and a threefold shorter half-life than R-modafinil, the effects of the racemic mixture are more similar to those of R-modafinil once steady state concentrations are reached, although small differences have been reported [35, 36]. Patients may benefit from one daily administration in the morning or from two administrations at morning and noon. A common side effect is headache (as with other stimulants), but this side effect generally subsides when doses are more slowly increased. Increased anxiety or blood pressure can also be observed. As for many other medications, modafinil can induce an allergic reaction, an effect that can escalate to a Stevens Johnson Syndrome (SJS) life-threatening situation. Because such an effect was suspected in a few cases during a pediatric trial of racemic modafinil, the compound is not approved for pediatric use [37]. The risk of a possible Stevens Johnson Syndrome with modafinil is to be balanced with that of an increased risk of addiction with more classic stimulants.

Modafinil is one of the few treatments that has been subjected to rigorous, double-blind, placebo-controlled studies in narcolepsy [24] and is approved by the Food and Drug Administration. Modafinil has also been approved for treating sleepiness in the context of shift work sleep disorder [38] and residual sleepiness in pressure airway therapy (PAP) or otherwise treated sleep apnea [39]. Because of the relatively low risk of addiction, modafinil can be more easily prescribed in patients without a clear, biochemically defined central hypersomnia syndrome, and is also easier to stop, if needed. It is also a schedule IV compound.

A typical treatment with modafinil (racemic mixture) in a typical adult hypersomnia/narcolepsy patient may start with 100 mg in the morning, and generally a second 100 mg dose will be added at noon. Then, the total dose may be increased to as much as 400 mg/day. For R-modafinil, as the potency is approximately twice, the initial dose may be 50 mg or 150 mg in the morning to increase to as much as 250 mg in the morning (equivalent to ~500 mg of the racemic mixture). Experience suggests that modafinil is effective for at least half of the patients, whereas in others it is simply too weak a stimulant. Slightly higher doses can be helpful in some patients, but are typically not covered by insurance. In such cases, we either would switch to other stimulants (methylphenidate) or use sodium oxybate and other treatment strategies. Of note, occassionally, it is helpful to add a dose of short-acting stimulant, such as methylphenidate (not slow-release) in addition to modafinil at times when alertness is particularly challenging (early afternoon), or as needed in case of an emergency (driving, etc.).

## Other Stimulants or Wake-Promoting Compounds

Unfortunately, most other DAT compounds available are either rarely used or have been withdrawn. These include pemoline (a low potency DAT inhibitor that was withdrawn due to dose-dependent hepatotoxicity) and mazindol (a high potency DAT inhibitor that also has additional properties making it difficult to increase the dose without experiencing side effects) [9, 31]. The antiviral amantadine and benztropine, an anticholinergic compound with DAT inhibitory effects used in Parkinson’s disease, are also notable. Higher affinity and more specific compounds, such as nomifensine (dual norepinephrine and dopamine reuptake inhibitor) and amineptine [29] have generally been withdrawn because of reports of abuse or misuse. Bupropion also deserves a brief mention, as it is a low potency nonspecific monoamine reuptake inhibitor that also has DAT inhibitory effects, probably explaining its stimulatory effects as an antidepressant. Selegiline was also previously mentioned.

Nondopaminergic wake-promoting compounds that are safe to use and efficacious are few. Caffeine, an adenosinergic antagonist, remains widely used, but has intolerable side effects at high doses (including cardiovascular), and it is generally not efficient enough for patients with hypersomnia or narcolepsy. Adrenergic reuptake inhibitors, such as atomoxetine [40, 41] or reboxetine (in Europe),which are compounds that have been developed for depression or ADHD, have a clear use in the therapeutic arsenal against narcolepsy and hypersomnia although undocumented by clinical trials (Table 1). These compounds increase wakefulness (but generally less strongly than DAT inhibitors, although there is great inter-individual variation) and they reduce cataplexy. We found these compounds to be very helpful in addition to (or instead of) modafinil in cases in which abuse could be an issue. Side effects include tachycardia and urinary retention, and increased anxiety in some individuals. Other modes of actions, such as histamine receptor H3 antagonists and hypocretin agonists, are being developed and will be discussed in the conclusion.

## Antidepressants as Anti-Cataplectic Agents

Reuptake inhibitor antidepressants generally do not have strong wake-promoting effects, although selective reuptake inhibitors with dual adrenergic serotoninergic effects can have some mild stimulant effects [42]. The use of these compounds is mostly reserved to treat cataplexy and other ancillary symptoms in narcolepsy. In animals and humans, reuptake inhibitors reduce REM sleep very potently after acute administration, likely explaining effects on cataplexy, a symptom with similarities to REM sleep atonia. Similarly, antidepressants also improve ancillary symptoms related to abnormal REM sleep, such as sleep paralysis and hypnagogic hallucinations.

A large number of antidepressants are active on cataplexy, including older compounds, such as tricyclic antidepressants [42]. Clomipramine, imipramine, desipramine, or protriptyline may all be used, although these compounds not only inhibit monoamine reuptake, they also have anticholinergic properties responsible for additional side effects (increased constipation, blurred vision, dry mouth, cardiac conduction impact). Other tricyclic antidepressants with antihistaminic and alpha-1 adrenergic blocking effects are also effective, but as they also induce sedation and orthostatic hypotension, they are rarely the best choice in narcolepsy.

New generation, selective serotoninergic (SSRI) or adrenergic reuptake inhibitors are generally preferred and are very effective. In narcoleptic canines, adrenergic reuptake inhibition has been shown to be the key property mediating anti-cataplectic effects for these compounds [29]. In humans, very selective serotoninergic reuptake inhibitors (e.g., escitalopram) are generally not as effective as dual serotoninergic-adrenergic reuptake compounds. For this reason, venlafaxine [43], or more rarely duloxetine or desmethylvenlafaxine, are commonly used and are probably the best first choice [41]. Unfortunately however, venlafaxine has a short duration of action so the extended-release form is preferable. With these compounds, doses inferior to those typically used for depression or anxiety treatment are generally sufficient. Venlafaxine, one of most used compounds, is typically started at 37.5 mg (extended release) in the morning, but is most often effective at doses of 75 to 150 mg extended release/day, although higher doses are needed in some patients.

Fluoxetine, a primary SSRI with some adrenergic effect via its metabolite desmethylfluoxetine, is effective, but higher doses, closer to the antidepressive effective doses (e.g., 20-60 mg/day) are needed. Fluoxetine, however, is occasionally useful when depressive or anxiety symptoms associated with narcolepsy are present, or when longer half-life is needed.

An advantage of antidepressant therapy is that it is immediately active on the symptom of cataplexy, unlike with depression or anxiety. A major problem, however, is rebound cataplexy with the cessation of treatment [44]. The effect is dramatic, it occurs the day after interruption of treatment (even with long-acting medication), and it may last for several weeks. For this reason, when patients are reporting the treatment to be ineffective, it is essential to check for compliance (or question the diagnosis of narcolepsy/cataplexy).

## Sodium Oxybate: A Compound with Beneficial Effects on All Narcolepsy Symptoms

Sodium oxybate, also called gamma hydroxybutyric acid (GHB), was first developed as an anesthetic agent. Unlike other anesthetic agents and sleep inducers, GHB was found to induce slow wave sleep and REM sleep, suggesting a very distinct mode of action and pharmacological profile. Based on this observation and considering the fact that many patients with narcolepsy/hypocretin deficiency have disturbed nocturnal sleep, the compound was tried with the hypothesis that increased sleep, notably REM sleep, would reduce sleep pressure during the day and allow narcoleptic patients to be more awake [45]. Numerous double-blind studies have now demonstrated that sodium oxybate is effective on many narcolepsy symptoms [46–50] and the compound is FDA approved for the treatment of cataplexy and excessive daytime sleepiness in narcolepsy. The compound also has additional documented effects on all other symptoms of narcolepsy (disturbed nocturnal sleep, sleep paralysis, hypnagogic hallucinations).

Because of its short half-life (30 minutes) [51] and a duration of action of only two to four hours, however, sodium oxybate typically needs to be administered twice during the night to fully consolidate a six to eight hour night. It is also a very low potency compound, requiring 6-9 g in adults per night to be effective (Table 1). Practically, in adults, we start with a 4.5 g dose split into two (one at bedtime and another in the middle of the night). We explain that the sedative effect is initially very strong, and that it is good to have someone else watch over the patient the first night, noting anything, such as snoring or gasping. Dizziness may occur, thus it is important to take the compound while in bed. We also explain that the unresponsiveness decreases somewhat as the patient becomes used to the medication. Patients are asked to call us back after the first day to give their impression, mostly for reassurance purposes. The dose is then increased to a total dose of 6 to 9 g, as 4.5 g is generally only partially effective, generally by 1.5 g steps every three to seven days, with weekly phone consultations.

Based on each individual’s response in terms of side effects (see as follows), and most importantly, their response in terms of duration of action on sleep and other symptoms, the dose is then adjusted not only quantitatively but also in its mode of administration. For example, we find that in many cases, the duration of action is not long enough to cover the entire night, such that a patient may take a 3 g dose at 10 PM to wake up at midnight, having to wait until 2 to 3 AM to take the take the second 3 g dose to fully sleep until 6 AM. In such patients, if we determine they have no difficulties falling asleep at 10 PM (exceptions would be in teenagers), we may suggest to first sleep without sodium oxybate for 1.5 h, then take the first dose at 11:30 PM to sleep until 2 AM, and then take the second dose and wake up at 6 AM. In rare cases, doses may even be split into three administrations, and may also be unequal in size. Our goal is to maintain close contact with the patient for the first few months of treatment to optimize the regimen so that nocturnal sleep is as good as possible, while maintaining the total amount required nightly within the active range (4.5 to 9 g), and also minimizing side effects.

Sodium oxybate is without any doubt one of the most effective medications available for narcolepsy/hypocretin deficiency. Not only does it have some effects on all symptoms, but a comparison of clinical trial effects on sleepiness, as measured subjectively (Epworth sleepiness scale) or objectively (MSLT or the Maintenance Test of Wakefulness (MWT), a variant of the MSLT most useful to assess whether patients can fight sleepiness) suggests that sodium oxybate is more effective than modafinil alone (400 mg) [24, 46]. The mode of action is unknown, but sodium oxybate is known to activate the Gamma-aminobutyric acid receptor type B (GABA-B receptor), an effect that has been shown to be essential in rodents for the mediation of sedation [52]. Other animal studies, however, suggest that the sleep effects of GHB may be different from those of baclofen [53]. In our opinion, because the compound has a very low affinity/low potency on the GABA-B receptor and has differential effects across species, it has been very difficult to demonstrate whether or not the GABA-B effect is sufficient for the therapeutic effects in narcolepsy. Baclofen, a prototypical GABA-B agonist, was used in narcolepsy and was found to be ineffective, suggesting that GABA-B alone may not be the entire story [54]. Unfortunately, however, baclofen has a longer half-life so it could still be that it is the combination of a very short-acting compound and GABA-B agonism that is responsible for the therapeutic effect.

A necessary area of investigation is whether or not optimizing nocturnal sleep with sodium oxybate, as previously described, is important for the full therapeutic effects in narcolepsy [55]. In one scenario, it is not, and then the effects on cataplexy and sleepiness are pharmacological and independent of sleep debt restoration. In the other scenario, sodium oxybate could act by reducing sleep debt so that when patients are waking up in the morning, they are so refreshed that they can stay awake much longer. Such a restorative effect would fit with the observation that the full therapeutic effect of sodium oxybate often takes weeks to months to manifest, not unlike the way sleep debt needs multiple days, if not weeks, to be fully recovered. One study also found correlations between increased slow wave sleep (SWS) activity and restorative effects after sodium oxybate in healthy subjects [56]. Because this is still unknown, and disturbed nocturnal sleep a clinical complaint on its own, our strategy always to try to optimize nocturnal sleep using sodium oxybate while also looking at the response of the other symptoms.

The use of sodium oxybate is limited by its side effect profile and the fact that it is difficult to prescribe. Regarding availability, prescription of sodium oxybate requires registration and training, and distribution to the patient is all made through a central pharmacy. The rationale for this decision was threefold. First, the compound has a very poor (but not necessarily deserved) public reputation. It is easily synthesized and has been used recreationally (e.g. to “cool down” after taking abused stimulants or MDMA) [57]. Second, as it is a strong sedative, it has also been used as a date rape drug [57]. Finally, under certain circumstances, such as continuous use at high dose, cessation can create severe withdrawal symptoms [58] and overdoses can be fatal, although in almost all cases fatal overdosing occurs in the context of polypharmacy.

In spite of this, however, in our experience that sodium oxybate is safe and efficacious when prescribed within the active dose range and when administration is limited to nighttime hours. When correctly prescribed, abrupt cessation does not lead to significant rebound or withdrawal effects, although a return to disturbed nocturnal sleep obviously occurs [59]. We certainly consider the compound first line for any patient with disturbed nocturnal sleep, cataplexy and obesity, and use it often in other cases. The most common side effects are nausea and weight loss [60]. Most of the time, these effects can be managed either by lowering the dose or by more progressively increasing the dose, or in very rare cases by adding anti-nausea medications, such as cyproheptadine or ondansetron. Rarely, however, nausea is so severe that administration is impossible.

Similarly, administration of sodium oxybate frequently leads to significantly decreased weight [60]. As narcolepsy/hypocretin deficiency is usually associated with weight gain (especially in children when the onset has been abrupt), weight loss is actually a very useful effect that allows a return to baseline. Weight loss, however, can be occasionally problemsome in patients who have not had weight gain and who have pre-existing anorectic tendencies. Another common side effect is enuresis, notably in younger patients, but this side effect is usually dose- and time-dependent, so that increasing the dose more slowly usually improves the situation. More rarely, parasomnias, such as sleep-walking, night eating (also common in untreated patients) or expiratory groaning [61, 62] can occur, and these side effects are also dose-dependent.

Other concerns have been raised and are not as well-established. First, sodium oxybate is a strong sedative, and as such there is the theoretical risk of increasing sleep-disordered breathing or hypoventilation (a common occurrence in obese narcoleptic subjects). Studies of the compound in sleep apnea patients have not found dramatic changes in apnea and hypopnea index, a measure of sleep apnea severity [63, 64], although the distribution of events by sleep stage was impaired. Others have raised the possibility that the effect is extremely variable and unpredictable, so that even some patients with mild sleep apnea could see their condition be exacerbated [65]. A controversy has even arisen on whether or not a prescription of sodium oxybate could be associated with a slightly increased death rate, a phenomenon that could not be assessed due to underreporting by the central pharmacy [66, 67]. A reanalysis of the data, however, does not suggest significantly increased mortality in subjects taking sodium oxybate [68].

To address this issue, we generally have a conservative common sense attitude so that patients with a high risk of sleep apnea or hypoventilation are monitored with polysmnography and CO_2_ monitoring while taking the drug. If there is significant disease, they are then treated with positive airway pressure (PAP) therapy first prior to the introduction of sodium oxybate, which paradoxically helps tolerate the PAP therapy in narcoleptic patients who can often have disturbed nocturnal sleep masked by sleep apnea [69]. Not uncommonly, we conduct titration of PAP under the influence of sodium oxybate, or restudy patients using polysomnography once a stable dose has been established. This is also systematically done if the response to sodium oxybate therapy on narcolepsy symptoms has not been as good as anticipated.

Second, clinical trials have shown that administration of sodium oxybate slightly increases anxiety ratings in many trials [70], although not always significantly, or in the pathological range. Other investigators have noted that in some rare cases reported as case reports, psychiatric complications may emerge, ranging from increased anxiety, to depression or even psychosis. Although not established, it is our experience that these effects are likely the result of increased daytime anxiety on a premorbid personality. For example, increased anxiety, depression, or a paradoxical feeling that the drug makes the sleepiness even worse can be the result of increased anxiety in a subject already pathologically anxious at baseline. Similarly, an individual with a paranoid personality may be biased toward having persecution delusions (similar to when stimulants are added). These effects are typically reversible once sodium oxybate is stopped, although it is not uncommon for us to then first add a course of SSRI to reduce anxiety and then reintroduce sodium oxybate with good results. Although these side effects are rare, this is an area in need of more research, as proper psychiatric screening is likely helpful to avoid potential problems if there is suspicion of psychiatric comorbidity. The situation is often complex in children and subjects close to onset, as in our experience a number of patients may develop narcolepsy together with behavioral disturbances secondary to sleepiness or a bona fide psychiatric disorder, such as schizophrenia, possibly the result of a larger autoimmune process.

## Therapeutic Approaches to KLS (Recurrent Hypersomnia, KLS Type)

No treatment has been shown to be clearly efficacious in KLS [71–73]. During episodes, patients are not only sleepy but cognitively impaired. Giving a stimulant can produce a paradoxical agitation, and thus is not indicated unless the episodes are mild (e.g., at a later phase of the disease when KLS is “burning out,” typically after 30 years of age).

In most cases, notably when episodes are not too frequent (e.g., 1-3 times/year) the best approach is to do no harm and to let the patient sleep through the episodes undisturbed, asking for accommodation at school, etc. Although depression is not a core feature of KLS, some patients can become extremely upset during episodes, and it is important to monitor mood. It is also important to make sure the patient does not leave the house unattended, driving or putting themselves in a dangerous situation during episodes. Reassurance regarding the long-term evolution of the condition is key.

In some cases, in which the episodes are severe and frequent (e.g., two weeks long, every 1-2 months), the only therapy that has been suggested to be efficacious in some cases is lithium, although there is controversy regarding its efficacy, with meta-analysis suggesting effects in 20 to 40 % of cases in preventing episodes and reducing severity [71, 72]. When lithium is introduced, it is important to raise the dose until adequate blood levels (0.8-1.2 mEq/ml) are attained [73]. In a few cases, lithium was added and removed, and clear effects on and relapses of lithium were observed, suggesting that the effect is real. The effects of other mood stabilizers, such as valproic acid or carbamazepine are less well-documented. Antidepressants are not efficacious. Working with a psychiatrist with experience using lithium is advised.

## Therapeutic Approaches in Narcolepsy/Hypocretin Deficiency *vs* Other Hypersomnias

At the pathophysiological level, it is now clear that most narcolepsy cases with cataplexy, and a minority of cases (5–30 %) without cataplexy or with atypical cataplexy-like symptoms, are caused by a lack of hypocretin (orexin) of likely an autoimmune origin. In these cases, once the disease is established, the majority of the 70,000 hypocretin-producing cells have been destroyed, and the disorder is irreversible. The cause is known and etiologically homogenous, and typically lifelong treatment will be needed, with the goal of optimizing life for these patients according to their goals. In our experience, as with any other disease entity, there is a range of symptom severity and variability in treatment response and side effects, but in approximately 80 % of cases, a return close to normal functioning is possible. A combination of lifestyle changes and pharmaceutical treatment tailored to each individual is typically needed.

Patients with narcolepsy hypocretin deficiency benefit best from combined drug therapy and behavioral modifications. Scheduled napping one to three times a day (with school or work accommodation often being needed) is very helpful to reduce the need for high dose stimulant. Driving and dangerous occupations must be discussed. In terms of drug therapy, we found that the majority of these patients do well on one to three of the following drugs: sodium oxybate, modafinil, and/or venlafaxine extended release. We tend to favor sodium oxybate as a first-line therapy, as in our experience this drug is at times sufficient by itself (especially in prepubertal children), whereas it is rare that a patient with narcolepsy/hypocretin deficiency will do optimally on modafinil or venlafaxine alone. Next line therapies may involve amphetamine-like stimulants, atomoxetine, or other antidepressants (added or in replacement, trying to limit the number of drugs).

For patients unlikely to have hypocretin deficiency, for example those negative for Human Leukocyte Antigen (HLA) DQB1*06:02 or/and without cataplexy, we are more cautious in terms of drug therapy for several reasons. First, the diagnosis of hypersomnia is difficult as it is a diagnosis of exclusion, made after other causes of daytime sleepiness (depression, sleep apnea, sedatives, disturbed nocturnal sleep, sleep deprivation, abnormal sleep cycle or shift work) have been ruled out. In clinical practice however, it is rare not to have some mild sleep apnea or some degree of disturbed sleep or mood disturbances in association with hypersomnia. It is thus hard to conclude whether the hypersomnia is purely primary and central. Second, the MSLT is known to have false positive results, even in “healthy” subjects who do not complain of daytime sleepiness [74, 75]. Third, whereas it is known that narcolepsy/hypocretin deficiency is a lifelong condition, little is known regarding the evolution of other hypersomnias of presumed central origin. Because of this, it is useful to challenge the notion of a lifelong therapy, and it is important to do no harm (e.g., establishing a stimulant addiction). Finally, clinical trials with modafinil and sodium oxybate have been performed in samples containing a majority of cases with cataplexy and presumably hypocretin deficiency, thus the response of these non hypocretin deficient cases is not as well-established.

In spite of these issues, it is unacceptable to dismiss these patients without providing treatment. Anemia, hypothyroidism, low iron status, infection (viral or Lyme disease), and general causes of fatigue must have been excluded. A careful evaluation of psychiatric status, sleep habits (during work and when on vacation), and sleep phase is essential, as is a sleep study to assess sleep apnea or other factors. The possibility that multiple factors could contribute should also be examined. The documentation of excessive sleep through logs or actigraphy is important. The MSLT is mostly useful to document objective daytime sleepiness, notably in the differential diagnosis of psychiatric issues, although it may be normal in severely affected patients who have extremely extended daytime sleep or cannot stay awake between naps.

Once the diagnosis is firm, it is essential to explain to the patient that the cause and evolution of the problem is unknown, and thus it is best to proceed cautiously. We generally advocate using low addiction potential medications, such as modafinil or atomoxetine first, and we have found that a portion of subjects react favorably. Regularizing sleep and wakefulness through behavioral interventions, such as mild sleep restriction and scheduled naps (as opposed to irregular 24-h sleep wake), can be helpful if the patient complies. Treating empirically with antidepressant is also worthwhile in some cases, especially when the MSLT is normal. Finally, recent findings suggest that some cases of unexplained hypersomnia are due to the accumulation of a GABA-enhancing natural compound in the brain, a pathology that could be reversed by flumazenil (briefly considering the short half-life) or the antibiotic clarithromycin (500 mg twice a day, with a maximum of 1 g/day) [76]. In this case, the positive effect of clarithromycin is secondary to a benzodiazepine antagonist-like effect, not its antibiotic effects, and treatment must be maintained.

Stronger drugs can also be tried, but if not helpful, should be rapidly withdrawn. Sodium oxybate can help significantly, notably if sleep difficulties are present, but special attention to psychiatric status is advised. Similarly, rare cases benefit from amphetamine-like stimulants, even at high doses, but in these cases we advise use of longlasting formulations to reduce the potential for addiction, and we recommend monitoring of the dose to a maximum of 60 mg/day, with a clear agreement by the patient that a higher dose will not be prescribed.

## Treatment of Children

The treatment of children with narcolepsy and hypersomnia is similar to that of adults with a few caveats. First, in children stimulants and antidepressants with adrenergic effects can reduce growth slightly [77]. Second, modafinil has not been FDA approved for use in children after reports of a few rashes, including one possible Stevens Johnson Syndrome in 1,500 cases [37]. Whether or not this report warrants no prescription *vs* surveillance is uncertain, and varies across countries.

In younger children (pre or peripubertal) with hypocretin deficiency, we have found that sodium oxybate can be uniquely effective (often at a dose close to the adult dose, even in younger children) reducing weight and normalizing sleep, wake, and cataplexy by itself [78–80]. The addition of one or two scheduled naps (or in a few cases adding low-dose stimulant and/or venlafaxine) is often sufficient to complement the therapy, although napping is often resisted by prepubertal children and needs gentle convincing. Failures to respond in this population are typically due to psychiatric side effects or when a psychiatric comorbidity has been developing concomitantly. Parental support is critical to proper adjustment of medication, with specificities due to age. For example, we occasionally use three lower doses of sodium oxybate in very young children so that the child can still sleep ten hours. In adolescent or older children, difficulties are often academic, requiring stronger treatment. Poor compliance due to behavioral issues can also be a problem.

## Pregnancy and Breast Feeding

This area is fraught by the absence of good human data, weak correspondence between animal and human teratogenicity, and the fact that classifications reflect length of exposure so that the longer a drug has been on the market, the more likely an issue may have been reported in humans. With this caveat in mind, amphetamines, methylphenidate, venlafaxine, and modafinil are category C (studies on animals show adverse effect and toxicity on fetus; no adequate and well-controlled studies done on pregnant women; drugs should be given only if the potential benefit outweighs the potential risk to the fetus). It should also be mentioned that modafinil has been suggested to interact with low-dose contraceptives, potentially reducing efficacy, although the scientific data supporting this claim is week and rests on poorly documented anecdotes. Sodium oxybate is category B (animal-reproduction studies have not demonstrated a fetal risk, but there are no controlled studies in pregnant women). These ratings suggest that there is no obvious teratogenicity, although it is impossible to exclude small developmental effects that could manifest later in life. As an example, animal studies have shown that rodents treated in pregnancy with antidepressants gave birth to animals with a behavioral profile consistent with depression. It is also clear that most of the drugs access the fetal brain, and in many cases, neonates may experience mild symptoms consistent with withdrawal.

The potential impact of any given drug varies depending on the pregnancy timing. For example, organogenesis begins three weeks after conception, so any problem before this timepoint would typically lead to a spontaneous abortion. Major teratogenic effects leading to birth defects and a viable child are likely to have occurred due to a problem occurring during the first trimester but after three weeks. Another critical period to consider is late pregnancy and delivery. As previously mentioned, mother and child may both be affected by psychotropic agents, and this may reduce vitality of the newborn and affect the ease of delivery.

At the practical level in one scenario a women may realize she is pregnant while still taking medications. In this case, reassurance is needed, as studies have shown that a large number, if not a majority, of pregnant women also report having taken some medication prior to realizing they were pregnant without any subsequent problem. In addition, as previously mentioned, there is no evidence that the drugs used in narcolepsy are notably teratogenic.

In a second scenario, a patient asks for advice for a planned pregnancy. In this case, we believe it is important to assess the potential guilt that could result if the patient kept using medications and a problem occurred. Work and family situations are also key. We generally suggest a conservative attitude and advise stopping the medications. Alternatively, careful high sensitivity monitoring of a potential pregnancy can be performed so that treatment is stopped as early as possible after conception, before organogenesis is initiated.

Later in pregnancy, it is also important to discuss delivery and the potential effects of the drugs on labor and on the immediate status of the newborn (see above), possibly stopping some medications prior to a planned delivery. Finally, when breastfeeding is initiated, it should be mentioned that all these psychotropic compounds can go into the breast milk. For these reasons, we advise using formula for a newborn if the patient wants to continue treatment.

## Conclusion and Emerging Therapies

Tremendous progress has been made in the treatment of narcolepsy with hypocretin deficiency (a pathology affecting 0.03 % of the population), although treatment remains symptomatically based. It is our opinion that in the majority of cases, functioning can be restored to approximately 80 % of normal, with the caveat that narcoleptic patients have to learn to adjust to their disability in the areas of wake and sleep, and be diligent with their medication and regularity in sleep/wake patterns. Difficulties are then most notable when an excessive amount of work is being required (e.g., at the end of high school or during stressful work situations).

In this area, it is likely that progress will come from two fronts. First, it is increasingly evident that narcolepsy is autoimmune and that an upper airway infection, maybe related to H1N1 influenza, triggers narcolepsy [81]. Furthermore, patients are diagnosed closer and closer to disease onset. Thus, it is now possible to explore whether immune modulation near disease onset could rescue the disorder if caught early enough. Trials using intravenous immunoglobulins in recent onset cases had mixed results, and not once was the disease fully reversed [82]. Interestingly, recent results suggest a T-cell rather than B-cell/antibody mediation [83]. Thus, it may be that trials with newer medications targeting T-cells (e.g., alpha-4 integrin inhibitors blocking T-cell entry to the brain, such as natalizumab) would have more beneficial effects. Progress in our therapeutic arsenal in this area, together with improved diagnostic methods as the autoimmune basis of narcolepsy becomes more understood, are likely to lead to preventive or therapeutic protocols in special cases.

Second, for established narcolepsy/hypocretin deficiency cases, the most logical intervention would be to use a hypocretin receptor agonist (dual, or selective for receptor 2) during the day. Central administration of hypocretin-1 reverses narcolepsy in animal models [84, 85], but unfortunately the hypocretin peptide does not cross the blood brain barrier, thus a centrally penetrating agonist is needed to be usable. Companies have successfully synthesized numerous molecules with hypocretin receptor antagonist properties [86], one of which is awaiting FDA approval for the treatment of insomnia; it is also likely that an agonist will be found and developed. Based on the fact that central administration of hypocretin-1 is strongly wake-promoting in rodents and dogs, it is likely that such compounds will be effective in narcolepsy and other hypersomnias. Whether or not they will have other desirable (e.g., antidepressant effects) or undesirable (addictive potential) effects will likely define their future therapeutic usefulness.

Finally, whereas a large number of safe hypnotics are available, clinicians have very few options for wake-promotion beside dopamine-acting compounds, such as modafinil and amphetamine-like stimulants. This is especially problematic for hypersomnia patients without hypocretin deficiency. Adrenergic reuptake inhibitors and caffeine can be used, but these only have mild stimulant effects. Benzodiazepine antagonist-like compounds were previously mentioned as potential therapies for a subset of hypersomnia patients, but they await confirmatory data. Companies have been developing H3 antagonists (i.e., compounds that promote the release of the wake-promoting amine histamine [87]), but whether or not these compounds will be particularly useful as wake-promoting agents in this population remain to be seen [88]. As approximately 1.5 % of the general population complains of excessive daytime sleepiness or excessive sleep amounts consistent with a hypersomnia disorder [89], we believe there is a strong therapeutic need for safe and effective compounds in this area.

## Electronic Supplementary Material

Below is the link to the electronic supplementary material.

ESM 1 (PDF 265 kb)
