# Supplementary material for: A Practical Guide to the Therapy of Narcolepsy and Hypersomnia Syndromes
Source: Neurotherapeutics. 2012 Oct 11;9(4):739–52. doi: 10.1007/s13311-012-0150-9 (PMC3480574; doi:10.1007/s13311-012-0150-9)
Supplement: Supplementary file 1 — (PDF 265 kb) [file 13311_2012_150_MOESM1_ESM.pdf]

ICMJE INTERNATIONAL COMMITTEE of MEDICAL JOURNAL EDITORS

ICMJE Form for Disclosure of Potential Conflicts of Interest

Section 1. Identifying Information

1. Given Name (First Name)
Emmanuel

2. Surname (Last Name)
Mignot

3. Effective Date (07-August-2008)
07-September-2012

4. Are you the corresponding author? ☑ Yes ☐ No

5. Manuscript Title
revised version of manuscript NERX-D-12-00110 entitled "A practical guide to the therapy of narcolepsy and hypersomnia syndromes".

6. Manuscript Identifying Number (if you know it)
NERX-D-12-00110

Section 2. The Work Under Consideration for Publication

Did you or your institution at any time receive payment or services from a third party for any aspect of the submitted work (including but not limited to grants, data monitoring board, study design, manuscript preparation, statistical analysis, etc…)?

Complete each row by checking "No" or providing the requested information. If you have more than one relationship click the "Add" button to add a row. Excess rows can be removed by clicking the "X" button.

The Work Under Consideration for Publication

Type	No	Money Paid to You	Money to Your Institution*	Name of Entity	Comments**	
1. Grant	☑	☐	☐			× ADD
2. Consulting fee or honorarium	☑	☐	☐			× ADD
3. Support for travel to meetings for the study or other purposes	☑	☐	☐			× ADD
4. Fees for participation in review activities such as data monitoring boards, statistical analysis, end point committees, and the like	☑	☐	☐			× ADD
5. Payment for writing or reviewing the manuscript	☑	☐	☐			× ADD
6. Provision of writing assistance, medicines, equipment, or administrative support	☑	☐	☐			×
